# MTHFR Gene-Polymorphism and Infertile Men in Indian Population: A Systematic Literature Review

**DOI:** 10.7759/cureus.27075

**Published:** 2022-07-20

**Authors:** Akash More, Ujwal Gajbe, Oluwabunmi Olatunji, Brij Singh

**Affiliations:** 1 Clinical Embryology, Jawaharlal Nehru Medical College, Datta Meghe Institute of Medical Science, Wardha, IND; 2 Anatomy, Datta Meghe Medical College, Datta Meghe Institute of Medical Science (DU), Nagpur, IND; 3 Clinical Embryology, School of Allied Health Science, Datta Meghe Institute of Medical Science (DU), Wardha, IND

**Keywords:** environment and lifestyle, male infertility, dna methylation, single nucleotide polymorphisms (snps), methylenetetrahydrofolate reductase (mthfr) gene

## Abstract

Single nucleotide polymorphisms (SNPs) in the genetic makeup of the methylenetetrahydrofolate reductase gene (*MTHFR* C677-T, A1298-C, and G1793-A) alongside environmental and lifestyle component has shown some links as a potential factor responsible for male infertility across the globe posing huge genetic vulnerability to the gender. However, SNPs in the *MTHFR* gene implicated in male infertility are not without their own controversial results even within the same population. The goal of this study was to provide comprehensive insights into the controversial nature of *MTHFR* gene polymorphism on male infertility across all Indian populations as well as other ethnicities. The electronic PubMed database was utilized to conduct and select eligible studies for this systematic review (update to December 2021). Only high-quality studies with a link between *MTHFR* polymorphisms and male infertility were included based on our exclusion and inclusion criteria. The connection between the *MTHFR* gene polymorphism and male infertility in Indian population studies was evaluated using odds ratios (ORs) with a 95% confidence interval (CI). A total of five studies presenting 1,237 cases and 1,044 controls were assessed for this study. The collective results revealed that *MTHFR *C667-T and A1298-C gene polymorphism were significantly linked with an increased chance of male infertility both in south India and north India, however, with some conflicting results. Interestingly, no study has been carried out to investigate the impact of G1793-A polymorphism on infertile males in the Indian population at the time of our report. Results generated from the few case-control evaluated on *MTHFR* gene polymorphism in the Indian population are found to conflict with some extrinsic factors (such as nutritional status-folate metabolism, lifestyle, varying recruitment procedures, and epigenetic elements) identified to have played some critical roles. Therefore, broader studies across all regions in India addressing the grave impact of *MTHFR* gene polymorphism on male infertility are of utmost importance.

## Introduction and background

Infertility is fast becoming a significant health issue worldwide. It is commonly described as the inability to reproduce within one to two years of unprotected consistent sexual intercourse [1]. Different types of infertility might be strongly affected by endocrine, systemic, epigenetic, environmental, or lifestyle factors. Continuous research has shown that males and females have equal chances of infertility rate of about 30%-40% [2].

Male infertility, a disorder with various complicated root causes, has been found to revolve around the way of life patterns, personal genetics, medical history, and environmental factors [3]. Medical researchers worldwide continue to see more connections between most diseases and genetic make-ups; infertility is no exception. Many genetic studies have connected congenital abnormalities/disorders and polymorphisms with male infertility [4]. These mutations or polymorphic activities in genes involved in spermatogenesis account for 15%-30% of male infertility [4]. Synthesis, methylation pattern, and repair of DNA, are essential gene mechanisms that enable proper and error-free genetic expressions; however, uniformity between the four deoxyribonucleotide triphosphate (dNTPs) is vital. This uniformity is regulated by the metabolism of Folic acid, leading to the stability and reliability of the gene [5]. And thus, any changes in the enzymatic route of this action may result in genetic/epigenetic dysfunctions [6].

Methylenetetrahydrofolate reductase (*MTHFR*), which is a critical enzyme in the metabolism of folate and interconversion of pyrimidines (uracil-thymine) during DNA synthesis, plays an essential role in balancing the methyl group in synthesis and methylation DNA [6]. *MTHFR* gene activity is significant in the vital process of spermatogenesis in adult testis [7]. *MTHFR* deficiency and folate insufficiency have demonstrated reactions that prevent the methylation of many substances, including proteins, RNA, DNA, and histones, due to a lack of methionine [8]. Therefore, diminishing activities of the *MTHFR* gene have been established to cause several disorders, including abnormal spermatogenesis and male infertility [8].

The effect of *MTHFR* anomaly on male infertility is yet to be fully understood, even though some studies have reported a link between *MTHFR* polymorphisms and infertility among Asian and Caucasian male populations with little or no reports from other human races, especially Africans [9]. More so, outcomes from published research on the correlation between the *MTHFR* gene-polymorphisms and male infertility remain conflicting due to varying reasons, including different research population selection processes and variances in genetics and environmental situations [10]. Based on this, this study aims to give thorough insights into the conflicting nature of *MTHFR* gene-polymorphism on male infertility in Indians and other races.

## Review

Literature search strategy

**Figure 1:**
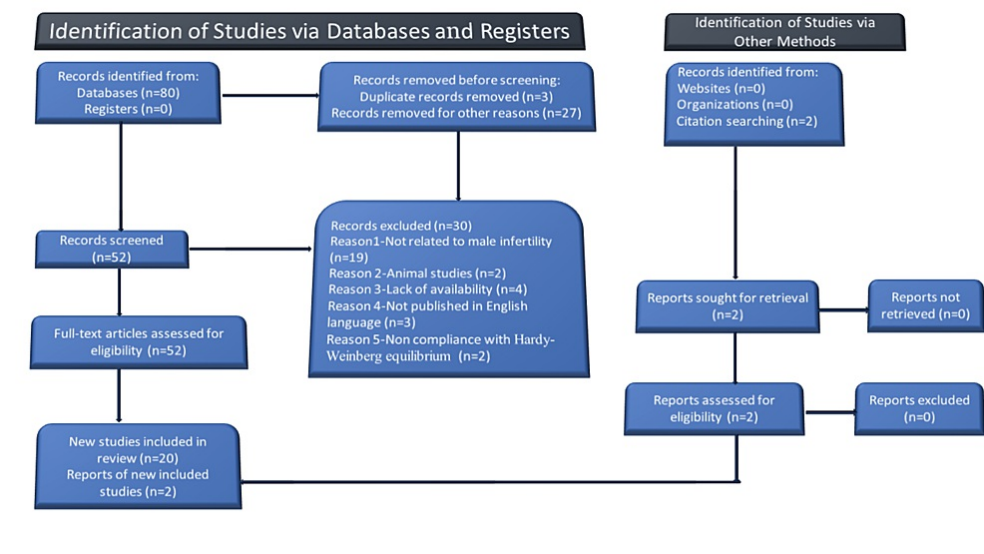
Flowchart of the article selection process.

We did a scientific investigation using PubMed and Google Scholar archives until December 2021. All published studies on the link between *MTHFR* gene polymorphism and male infertility published up to December 2021 were reviewed, and the most related outcomes were included. This search was conducted using the following keywords; Methylenetetrahydrofolate reductase (*MTHFR*), Single Nucleotide Polymorphism (SNP), genes, DNA methylation, and male infertility. Also, the references from extracted articles were assessed to find other qualified studies that were omitted by the search. Figure 1 shows the search mechanism.

Eligibility criteria

Inclusion factors for study eligibility involve: 1) Relevant *MTHFR* C677-T, A1298-C, or G1798-A polymorphisms and male infertility studies. 2) Human case-control design assessing at least one of the SNPs. 3) Presence of the three variant genotypes of either *MTHFR* C677-T or A1298-C polymorphisms. 4) Research indicated the frequency of the *MTHFR* C677-T, A1298-C, or G1798-A polymorphisms. 5) Publication in English. 6) Availability of full-text article.

Exclusion factors for ineligibility were: 1) No relation to the *MTHFR* C677-T, A1298-C, or G1798-A polymorphisms and male infertility. 2) Absent of usable or adequate genotype data reported. 3) Animal study, conference abstract, editorial article, and meta-analysis. 4) Studies that did not follow Hardy-Weinberg equilibrium. 5) Related articles published in other languages.

Data extraction

All recruited articles were assessed by reading the full text per availability and analyzed further. The “Preferred Reporting Items for Systemic Review and Meta-Analysis (PRISMA) Standards” were used to search for this systemic review (Figure 1). A third author verified the extracted data by entering it into a collection form. Discussion and consensus were used to resolve disagreements. Name of the first author, year of publication, area, genotyping method, and case and control group sample sizes were retrieved for each research.

Result

The PubMed research portal searched “*MTHFR*” and found 7,984 articles, while the keyword “*MTHFR* gene polymorphism” yielded 5,064 articles. The PubMed online database searched for all articles on the effect of *MTHFR* gene- polymorphism on male infertility. *MTHFR* gene polymorphism on male infertility showed 82 results. After removal of all articles with the exclusion criteria, a total number of 22 studies were added in this systematic review.

Therefore, this systematic review pooled together data from 22 published studies out of which five were case-control studies from India with 1,237 cases and 1,044 controls for C677-T and A1298-C *MTHFR* gene polymorphism without any study on the G1793-A polymorphism. While the remaining 17 studies showed various results from the three SNPs. Similarly, we studied the results outcomes from other populations with different variations. some studies from Pakistan, Iran, Jordan, South Korea, Brazil and Turkey all reported relevant connections between *MTHFR* C677-T polymorphism and male infertility. Other studies from Morocco, South Korea, Brazil, concluded that *MTHFR* A1298C polymorphism affects male infertility and studies from Iran showed a link between the third polymorphism (G1793-A) and male infertility. Contradictory to this, different studies from Algeria, Poland, France, Italy, and China stipulated no relationship between the three SNPs and infertility in males.

Discussion

Variations in genetic makeup alongside environmental and lifestyle components have been proposed to be linked with male infertility. *MTHFR* gene is a likely potential cause for genetic vulnerability as mutations and some polymorphisms can cause a decrease in the activities of MTHFR enzymes and, ultimately, spermatogenic failure and infertility [11]. *MTHFR*, which is a significant participant in the regulatory enzymes of folate metabolism, helps in the synthesis, re-methylation of DNA, and in vivo control of homocysteine levels [12]. This enzyme triggers the reduction of 5-10 methylenetetrahydrofolate (5, 10-methyl THF) to 5 methyltetrahydrofolates (5-methyl THF), a donor of methyl in the re-methylation of homocysteine to methionine [12]. On the other hand, methionine is converted to S-adenosylmethionine (methyl donor) utilized in quite a few reactions through which methylation of substrates- DNA, RNA, hormones, and lipids occurs [13]. Consequently, due to the importance of this enzyme during DNA activities, any alterations in its sequence might affect spermatogenesis, resulting in infertility [14].

The *MTHFR* gene is made up of 11 exons situated at the short arm of chromosome 1 (1p36.3) [13,14]. There are three different types of variations at a single position in the DNA sequence of the *MTHFR *gene termed - SNPs that ultimately affect the activities of these enzymes [15]; these include C677-T, A1289-C, G1793-A (8,11). Based on previous reports, C677-T and A1298-C polymorphic variants are the most frequent variation in the *MTHFR* gene [14]. Any form of this mutation at any point can lead to thermal instability and reduced activities of MTHFR enzymes [15].

MTHFR C677-T

This is the most seen and observed SNP in the *MTHFR* gene [16]. During polymorphism of this gene, there is C-to-T movement at nucleotide 677 in exon 4 points where cytosine (C) is mutated to a thymine (T) at the coding region *MTHFR *gene in humans, resulting in an alanine-valine mutation where alanine position is replaced with valine residues (Ala222Val) consequently, generating a decrease in enzyme activity [17]. This leads to a phenotype high in homocysteine and readily deactivated by heat. Several pieces of research have reported various connections between *MTHFR *C677-T polymorphism and male infertility in different populations, further saying that the MTHFR enzyme's specific activity is lowered by 35% in the C-T genotype compared to C-C normal genotype and by 70% in the T-T genotype [18,19].

MTHFR A1298-C

This is another common SNP in the *MTHFR *gene [20]. The polymorphism of the *MTHFR *A1298-C gene results in the mutation of cytosine-adenine in exon 7 points of the gene, where glutamate position is replaced with alanine (Glu429Ala) [21]. Similarly, it reduces biochemical activities of the MTHFR enzyme - to a lesser degree than *MTHFR *C677-T. Although it is a famous SNPs-*MTHFR *gene, it is the most controversial among various ethnicities [22]. Compared to *MTHFR *C667-T, the A1298-C form of *MTHFR *has been investigated less extensively globally [23]. Despite the thermostability of *MTHFR *A1298-C variation, in vitro investigations have revealed that it has 65% of wild-type enzymatic activity, compared to 40% for the C667-T variant [24,25].

MTHFR G1793-A

This is the third polymorphic site of the *MTHFR *gene [26]. It is a novel site that has received minimal attention the most. The G1793-A polymorphism occurs with the exchange of arginine with glutamine at codon 594 (Arg594Gln). In some studies, it was described to play some roles in male infertility. The survey by Safarinejad et al. reported that the combined 677C-T and 1793G-G and 677T-T and 1793G-G variants were linked to an increased risk of infertility [11,26]. However, some studies have also found no connection between this polymorphism and male infertility [5,27].

Folate metabolism has some important weight on normal and abnormal functions in most aspects of reproductive medicine [28]. In support of this, continuous studies keep demonstrating the link between folate deficiencies, hyperhomocysteinemia, gonadal disorders and infertility [29]. Similarly, several experimental outcomes have reported that enzymes involved in the folate pathway are essential for spermatogenesis and mutations in the genes of these enzymes particularly, *MTHFR* along with folic acid shortage can disrupt nucleotide synthesis and may lead to infertility [30]. Reduced *MTHFR* enzyme activity in sperm cells is known to be caused by mutated *MTHFR* genes, resulting in lower methionine availability and DNA methylation [31,32]. Hence, low DNA methylation may cause decreased activity of the *MTHFR* gene in sperm cells which may lead to low folate metabolism and ultimately low male infertility rates [33]. However, different polymorphisms of this gene from the diverse population have displayed different outcomes relating to male infertility [33].

Out of the five studies from India, three of them showed no link between *MTHFR* gene-polymorphism and infertile males in India, while the remaining two suggested a relationship between *MTHFR* gene-polymorphism and male infertility [34-38]. However, only one study showed an association between A1298-C polymorphism and male infertility out of two studies conducted [36-38]. Furthermore, three out of the five studies reported results from the north Indian male population (Table 1) [34-36]. The first finding by Naqvi et al. which addresses the effect of SNPs-*MTHFR* C677-T polymorphism on male infertility reported a significant correlation suggesting it may serve as a genetic cause for infertility in males [34]. The second which was conducted by Singh et al. concentrated only on *MTHFR* C677-T polymorphism also reporting that this particular gene mutation affects the Indian population [35]. Thirdly, Dhillon et al. in 2007 reported no correlation between this gene-polymorphism and male infertility [37,38]. This study was also the only study from North India that researched the involvement of *MTHFR* A1298-C polymorphism on male infertility yet, reporting no association with male infertility in this population [36].

**Table 1:** Results of MTHFR gene polymorphism on male infertility in the Indian population. Methylenetetrahydrofolate Reductase (MTHFR), Single Nucleotide Polymorphism (SNP).

AUTHOR	MTHFR C677-T	MTHFR A1298-C	MTHFR G1793-A	SUMMARY
Vani et al. (2011) [37]	No association	Not included	Not included	This study focused on the South Indian population where it reported the absence of any association of MTHFR C677-T gene-polymorphism with male infertility.
Naqvi et al. (2013) [34]	Associated	Not included	Not included	This study focused on the North Indian population where it reported the correlation of MTHFR C677-T gene-polymorphism with male infertility.
Singh et al. (2005) [35]	Associated	Not included	Not included	Similarly, this study agrees with Naqvi et al. that MTHFR C677-T gene-polymorphism is a genetic factor that contributes to infertility in the North Indian population.
Balunathan et al. (2021) [38]	No association	Associated	Not included	In line with Vani et al., this study also found no relationship between MTHFR C677-T gene-polymorphism and infertility in South-Indian men. However, reported that there is a link between MTHFR A1298-C and male infertility
Dhillon et al. (2007) [36]	No association	No association	Not included	Although from the North Indian population, this study found no association between any of the SNPs, except when CT and TT (C677-T) taken together were combined.

**Figure 2:**
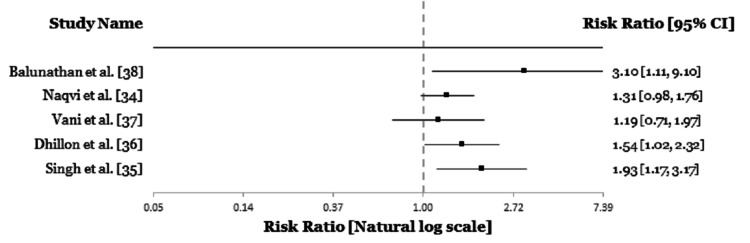
Forest plot showing the link between MTHFR C677-T polymorphism and male infertility in Indian population.

Similarly, two studies (Vani et al. and recently, Balunathan et al.) from the South Indian population, found no link between *MTHFR* C677-T polymorphism and male infertility [37,38]. Although Vani et al. did not carry out any study on the A1298-C polymorphism on infertility in males, Balunathan did and found a relevant association between *MTHFR* A1298-C polymorphism and infertility [38]. To present, there is no study from India showing any relationship between *MTHFR* G1793-A polymorphism on infertile in India (Table 2, Figure 2).

**Table 2:** Characteristics of an Indian population study. Polymerase chain reaction (PCR), Restriction fragment length polymorphism (RFLP)

Author (year)	Patients (men)	Method	HWE
Vani et al. (2011) [37]	206- cases 230- control	PCR\RFLP	-
Naqvi et al. (2013) [34]	637- cases 364- control	PCR\RFLP	-
Singh et al. (2005) [35]	165- cases 200- control	PCR\RFLP	Yes
Balunathan et al. (2021) [38]	50- cases 50- control	PCR\RFLP	Yes
Dhillon et al. (2007) [35]	179- cases 200- control	PCR\RFLP	Yes

Furthermore, we summarized outcomes from other populations with different variations. Nine studies from Pakistan, Iran, Jordan, South Korea, Brazil and Turkey all reported relevant connections between *MTHFR* C677-T polymorphism and male infertility [39-47]. While three studies from Morocco, South Korea, Brazil, concluded that *MTHFR* A1298C polymorphism affects male infertility [46,48,49]. Also, two studies from Iran showed a link between the third polymorphism and male infertility [41,50] while three studies from Iran, France and Brazil found no significance between these two [41,45,51]. Contradictory to this, five different studies from Poland, Italy, Algeria, China and France stipulated no relationship between the three SNPs and infertility in males [51-55].

**Figure 3:**
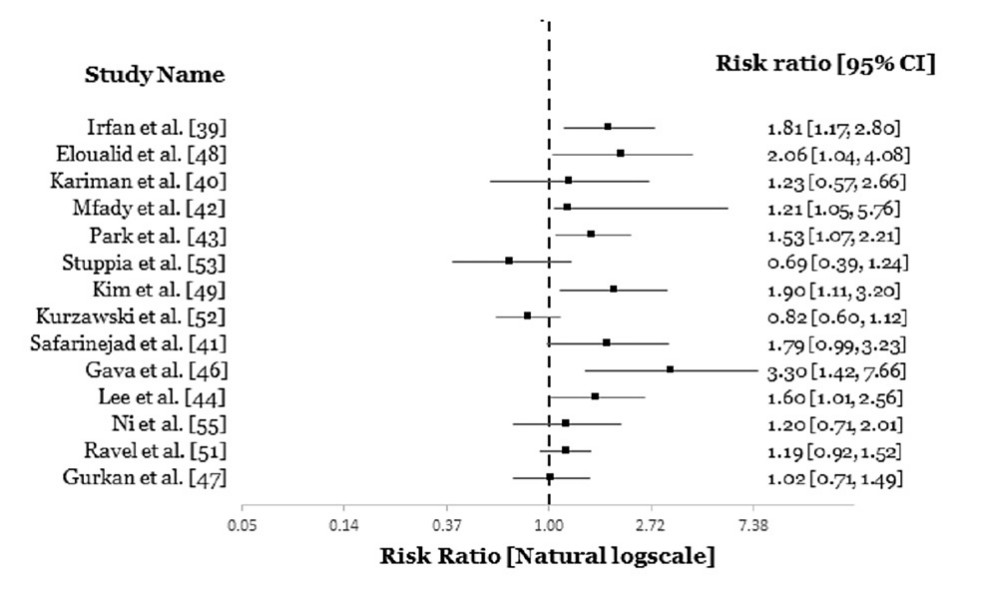
Forest plot showing the link between MTHFR C677-T polymorphism and male infertility in other populations.

**Figure 4:**
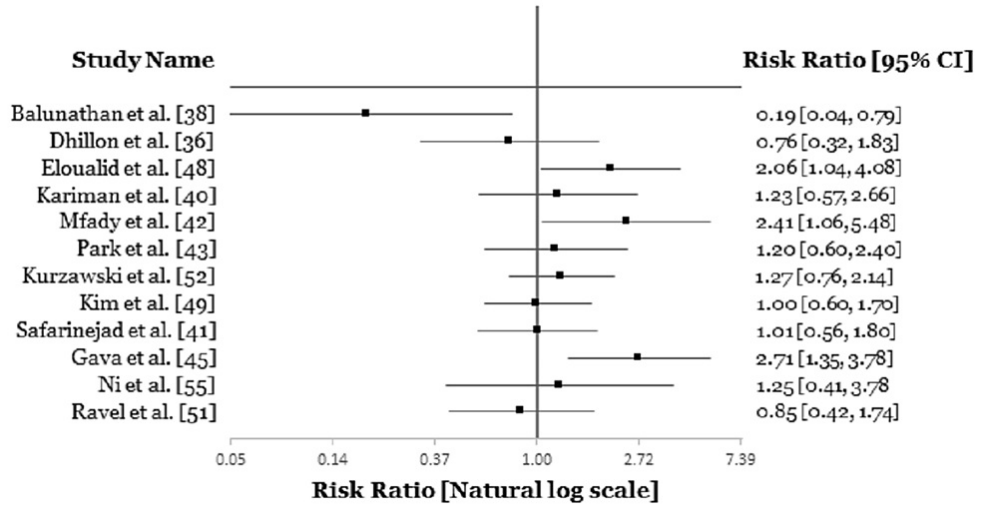
Forest plot showing the link between A1298-C polymorphism and male infertility.

Based on the above mentioned it is clear that the impact of *MTHFR* gene polymorphism is very controversial and this may be due to variations in nutritional status (folate metabolism), lifestyle, varying recruitment procedure and epigenetic factors (Table 3, Figures 3, 4) [56]. Some of the limitations we observed were the small sample size and the combined study on sperm abnormalities parameter. To overcome this, a larger sample size should be used, and individual studies should be carried out on the impact of *MTHFR* gene mutation on each abnormal sperm parameter. This would give better clarity into the degree to which this gene polymorphism affects a specific population of people and the role it plays in each abnormal sperm parameter.

**Table 3:** Reports of the impact of MTHFR gene polymorphism on male infertility in other locations.

Author (year)	Location	MTHFR C677-T	MTHFR A1298-C	MTHFR G1793-A	Remark
Irfan et al. (2006) [39]	Pakistan	Associated	Not included	Not included	
Kariman et al. (2017) [50]	Iran	Not included	Not included	Associated	
Eloualid et al. (2012) [48]	Morocco	Not associated	Associated	Not included	
Kariman et al. (2014) [40]	Iran	Associated	Not associated	Not included	
Mfady et al. (2013) [42]	Jordan	Associated	Not associated	Not included	
Park et al. (2005) [43]	South Korea	Associated	Not associated	Not included	
Stuppia et al. (2003) [53]	Italy	Not associated	Not included	Not included	
Kurzawski et al. (2015) [52]	Poland	Not associated	Not associated	Not included	
Kim et al. (2015) [49]	South Korea	Not associated	associated	Not included	
Saferinejad et al. (2011) [41]	Iran	Associated	Not associated	Not associated	However, the combination of all three variants showed an association with male infertility
Gava et al. (2011) [46]	Brazil	Associated	Not associated	Not associated	Combined showed no association
Gava et al. (2011) [45]	Brazil	Associated	Associated	Not included	
Lee et al. (2006) [44]	South Korea	Associated	Not included	Not included	
Ni et al. (2014) [55]	China	Not associated	Not associated	Not included	
Chellat et al.(2011) [54]	Algeria	Not associated	Not included	Not included	
Ravel et al. (2009) [51]	France	Not associated	Not associated	Not associated	
Gurkanet.al. (2014) [47]	Turkey	Associated	Not included	Not included	

## Conclusions

The effect of *MTHFR* gene-polymorphism on male infertility is yet to be fully understood as a result of the many discrepancies in the results obtained from various studies from different locations and ethnicities. India's population is not an exception to these differences. In this study, we looked at all the studies carried out on this subject matter on the Indian population, and we found only seven related studies. Out of these, five were original articles, while two were meta-analyses. Mainly, these studies were carried out on men from North and South India with controversial outcomes. However, studies that report the activities of *MTHFR* gene-polymorphism on male infertility in the West and East population of India are yet to be written. Also, there is a lack of data on India's third *MTHFR* gene polymorphism (G1793-A). Therefore, there is a need for further intensive study on the positive or negative weight of all the three *MTHFR* gene polymorphisms on male infertility across all Indian populations, promoting the need for a better understanding of these mutations and the application of proper medical management and treatment.
